# Fatigue in Sjögren's Syndrome: A Search for Biomarkers and Treatment Targets

**DOI:** 10.3389/fimmu.2019.00312

**Published:** 2019-02-26

**Authors:** Iris L. A. Bodewes, Peter J. van der Spek, Leticia G. Leon, Annemarie J. M. Wijkhuijs, Cornelia G. van Helden-Meeuwsen, Liselotte Tas, Marco W. J. Schreurs, Paul L. A. van Daele, Peter D. Katsikis, Marjan A. Versnel

**Affiliations:** ^1^Department of Immunology, Erasmus MC, University Medical Centre Rotterdam, Rotterdam, Netherlands; ^2^Department of Bioinformatics, Erasmus MC, University Medical Centre Rotterdam, Rotterdam, Netherlands; ^3^Department of Pathology, Erasmus MC, University Medical Centre Rotterdam, Rotterdam, Netherlands; ^4^Department of Internal Medicine, Erasmus MC, University Medical Centre Rotterdam, Rotterdam, Netherlands

**Keywords:** Sjögren's syndrome, fatigue, interferon, proteomics, SOMAscan

## Abstract

**Background:** Primary Sjögren's syndrome (pSS) is a systemic autoimmune disease, where patients often suffer from fatigue. Biological pathways underlying fatigue are unknown. In this study aptamer-based SOMAscan technology is used to identify potential biomarkers and treatment targets for fatigue in pSS.

**Methods:** SOMAscan® Assay 1.3k was performed on serum samples of healthy controls (HCs) and pSS patients characterized for interferon upregulation and fatigue. Differentially expressed proteins (DEPs) between pSS patients and HC or fatigued and non-fatigued pSS patients were validated and discriminatory capacity of markers was tested using independent technology.

**Results:** Serum concentrations of over 1,300 proteins were compared between 63 pSS patients and 20 HCs resulting in 58 upregulated and 46 downregulated proteins. Additionally, serum concentrations of 30 interferon positive (IFNpos) and 30 interferon negative (IFNneg) pSS patients were compared resulting in 25 upregulated and 13 downregulated proteins. ELISAs were performed for several DEPs between pSS patients and HCs or IFNpos and IFNneg all showing a good correlation between protein levels measured by ELISA and relative fluorescence units (RFU) measured by the SOMAscan. Comparing 22 fatigued and 23 non-fatigued pSS patients, 16 serum proteins were differentially expressed, of which 14 were upregulated and 2 were downregulated. Top upregulated DEPs included neuroactive synaptosomal-associated protein 25 (SNAP-25), alpha-enolase (ENO1) and ubiquitin carboxyl-terminal hydrolase isozyme L1 (UCHL1). Furthermore, the proinflammatory mediator IL36a and several complement factors were upregulated in fatigued compared to non-fatigued pSS patients. ROC analysis indicated that DEPs showed good capacity to discriminate fatigued and non-fatigued pSS patients.

**Conclusion:** In this study we validated the use of aptamer-based proteomics and identified a novel set of proteins which were able to distinguish fatigued from non-fatigued pSS patients and identified a so-called “fatigue signature.”

## Introduction

Primary Sjögren's syndrome (pSS) is a common systemic autoimmune disease, characterized by lymphocytic infiltrations in salivary and lachrymal glands. This is accompanied by sicca symptoms of the eyes and mouth and frequently also extraglandular manifestations (1–3). Fatigue is one of the most common extraglandular manifestation in pSS and is associated with a poor quality of life (4–9). Fatigue affects up to 70% of pSS patients, while ~20% of healthy adults are affected (10–13).

The biological basis of fatigue is largely unknown, however proinflammatory mechanisms are thought to play a role. Interferons (IFNs) are proinflammatory cytokines, which play a pivotal role in the pathogenesis of pSS and are systemically upregulated in 57% of the pSS patients (14). Elevated levels of IFNs induce increased expression of IFN-stimulated genes in the salivary glands, peripheral blood mononuclear cells (PBMCs), isolated monocytes and B cells of pSS patients (15–19). This so-called “IFN type I signature” is associated with higher disease activity and higher levels of autoantibodies. In addition, mutations affecting IFN signaling are observed in TREX, IRF5, STAT4, and PTPN22W and are associated with pSS (20–26). There is evidence for a link between IFNs and fatigue. Patients receiving IFNα treatment for viral hepatitis or melanoma can develop severe fatigue (27, 28). However, we and others have previously shown that there was no correlation between systemic upregulation of IFNs and fatigue in pSS patients (13, 29).

Because fatigue is a common problem in pSS, it is important to identify pathways underlying this fatigue. Here we use a proteomics approach to identify pathways related to fatigue. We used the aptamer-based SOMAscan technology, a highly multiplexed proteomic assay that queries 1,300 proteins in serum for protein biomarker discovery and identification of serum proteomic signatures and possible treatment targets for fatigue in pSS.

## Methods

### Patients and Methods

PSS patients and healthy controls (HC) were recruited at the Erasmus Medical Centre, Rotterdam, the Netherlands. All pSS patients fulfilled the 2002 American-European Consensus Group classification criteria (30) and were free of symptoms of viral infection at inclusion. HC did not suffer from autoimmune disease and did not use corticosteroids. Written informed consents were obtained from all participants in the study, in compliance with the Helsinki Declaration. Medical Ethical Review Committee of the Erasmus MC approved this study.

### Blood Collection

Blood was collected in clotting tubes for serum preparation and in PAXgene RNA tubes (PreAnalytix, Hombrechtikon, Switzerland) for whole blood RNA analysis.

### Real-Time Quantitative PCR and Calculation of IFN Score

Total RNA was isolated from PAXgene tubes and reverse-transcribed to cDNA. For calculation of relative expression, samples were normalized to expression of the household gene Abl [31]. Relative expression values were determined from normalized CT values using 2^∧^-ΔΔCT method (User Bulletin, Applied Biosystems). The IFN score was defined by the relative expression of 5 genes, IFI44, IFI44L, IFIT1, IFIT3, MXA. Mean_HC_ and SD_HC_ of each gene in the HC-group were used to standardize expression levels. IFN scores per subject represent the sum of these standardized scores, calculated as previously described (31, 32). Patients were divided in groups being positive or negative for the IFN score using a threshold of Mean_HC_ + 2 x SD_HC_.

### Proteomic Analysis of Serum Protein Concentrations

Serum protein concentrations were measured using the SOMAscan platform. SOMAscan utilizes single stranded DNA-based protein affinity reagents called SOMAmers (Slow Off-rate Modified Aptamers) (33, 34). The SOMAscan 1.3k kit was used following manufacturer's protocol, measuring over 1,300 proteins in 65 μl of serum. Intra-run normalization and inter-run calibration were performed according to SOMAscan assay data quality-control procedures as defined in the SomaLogic good laboratory practice quality system. Data from all samples passed quality-control criteria.

### Measurement of Complement, Immunoglobulin Levels, and Autoantibodies

C3, C4, and IgG were measured as described previously (14). Anti-SSA and anti-SSB were determined by EliA (Thermo Fisher Scientific), confirmed with ANA profile immunoblot (EuroImmun) and re-confirmed where needed by QUANTA Lite ELISA-kit (INOVA).

### Assessment of Fatigue and Depressive Symptoms

Fatigue was assessed using the Dutch version of the multidimensional fatigue inventory (MFI) (35). As a cut-off the 25 percentile highest (fatigued group) and lowest scores (non-fatigued group) were used resulting in the inclusion of 45 patients. The Dutch-validated Center for Epidemiologic Studies Depression (CES-D) was used to study depression and anxiety (36, 37).

### Statistics

SOMAscan was performed to identify differences in quantitative binding of proteins to aptamers. Data were analyzed using empirical Bayes moderate *t*-test by the limma Bioconductor package in the R environment (38–40). *P*-values were corrected for multiple hypothesis testing using Benjamini-Hochberg method (FDR < 0.05). Differential expression was calculated on normalized log_10_ intensities. Visualization of differentially expressed proteins (DEPs) between pSS and HC and fatigued and non-fatigued pSS patients was based on ^2^Log transformed binding intensities and geometric means were calculated for HC and pSS patients.

Independent *T*-test was used to compare means and the Mann-Whitney U test was used to compare medians. Categorical data were compared using Fisher's exact test and correlations were assessed using Spearman's rho (r_s_). In order to determine the discriminatory capacity of markers receiver operating characteristics (ROC) curves and areas under the curves (AUCs) were calculated. Statistical analysis and visualization of DEP was performed using Instem/Omniviz, R version 3.4.3 bioconductor package limma version 3.34., IBM SPSS 24.0 (SPSS, Chicago, IL, USA) and Graphpad Prism 5.0 (Graphpad Software, La Jolla, CA, USA), 9.

## Results

### Differential Protein Expression in Serum of pSS and Interferon Positive Patients

**Figure 1:**
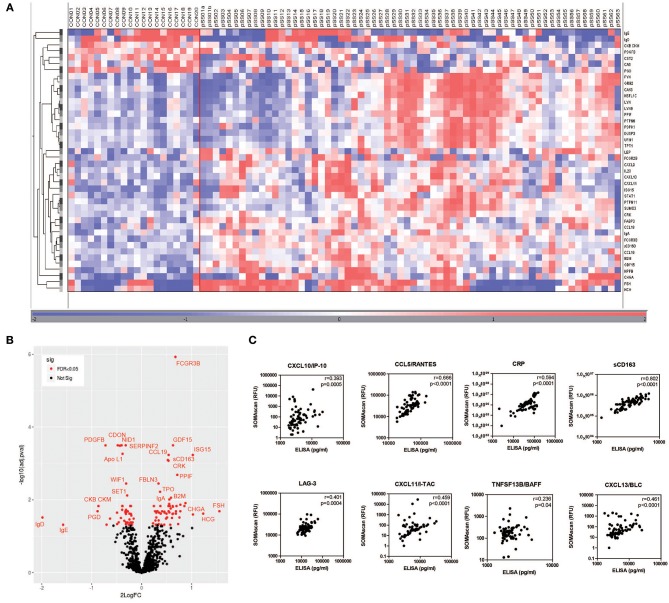
Differential protein expression in serum of pSS and healthy controls. Heatmap of differentially expressed proteins measured by SOMAscan technology in serum samples of pSS patients (pSS) (*n* = 63) and healthy controls (CON) (*n* = 20) clustered unsupervised within the groups **(A)** and volcano plot **(B)** visualizing the same DEPs. The correlation between RFU determined by SOMAscan and protein levels determined by ELISA is shown in **(C)**.

Characteristics of pSS patients and HC are summarized in Supplementary Table 1. Using the SOMAscan multiplex proteomic assay, in total 104 serum proteins were differentially expressed between pSS patients and HCs after correction for multiple testing. Of these proteins 58 were upregulated and 46 were downregulated. A heatmap representing the most significant DEPs (^2^LogFC>0.5) is shown in Figure 1A and indicates a clear distinction between pSS and HCs (Figure 1A). Figure 1B shows in a volcano plot for the same DEPs. Top upregulated DEPs include Fcγ receptor 3B, a receptor binding immune complexes which are often observed in pSS, the interferon-inducible protein ISG15 and several female hormones including follicle-stimulating hormone (FSH) and human chorionic gonadotropin (HCG). A complete list of all significantly up- and downregulated proteins is shown in Supplementary Table 2.

Additionally, IFN positive (IFNpos) and IFN negative (IFNneg) pSS patients were compared. Characteristics of IFNpos and IFNneg pSS are summarized in Supplementary Table 3. IFNpos patients showed significantly higher IgG levels, higher frequency of anti-SSA and anti-SSB autoantibodies and lower C3 complement levels compared to IFNneg patients. Comparing protein expression between IFNpos and IFNneg patients 38 proteins were DEPs of which 25 were upregulated and 13 were downregulated. As expected many interferon-inducible proteins were found upregulated including ISG15, CXCL11/I-TAC, CXCL10/IP-10, OAS1, and TNFSF13B/BAFF. Volcano plots and a full list of all significantly up- and downregulated proteins between IFNpos and IFNneg pSS patients is shown in Supplementary Figure 1 and Supplementary Table 4.

To validate the SOMAscan data, ELISAs were performed for several DEPs between pSS patients and HCs or IFNpos and IFNneg pSS patients. Proteins for validation were selected based upon availability of reliable ELISAs. The selected proteins included CXCL10/IP-10, CCL5/RANTES, CRP, sCD163, LAG-3, CXCL11/I-TAC, TNFSF13B/BAFF, and CXCL13/BLC. All protein levels measured by ELISA significantly correlated with relative fluorescence units (RFUs) determined by the SOMAscan (Figure 1C).

### Fatigued pSS Patients Are Characterized by a Differential Serum Protein Expression Pattern

**Figure 2:**
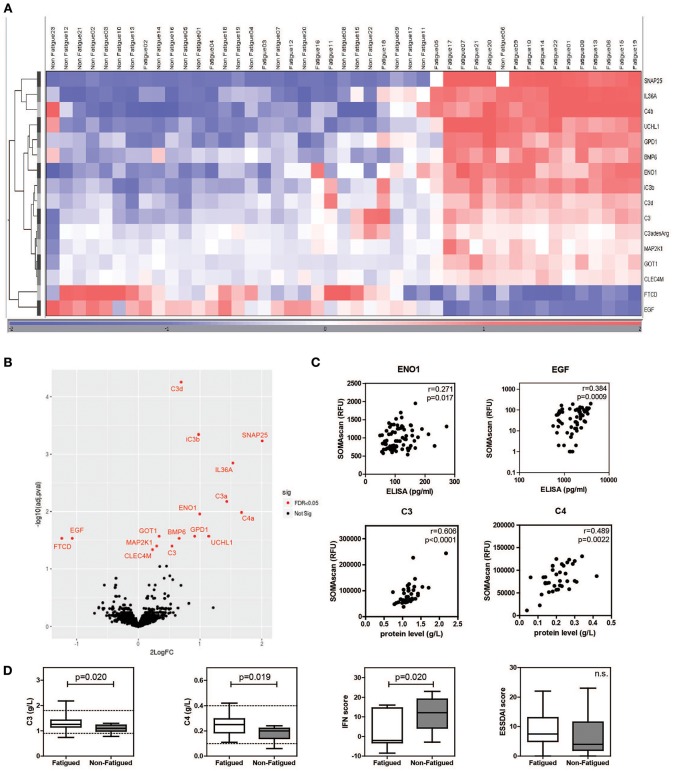
Differential protein expression in serum of fatigued pSS patients. Heatmap showing the unsupervised clustering of differentially expressed proteins between fatigued (*n* = 22) and non-fatigued (*n* = 23) pSS patients **(A)** and volcano plot **(B)** visualizing the same DEPs. **(C)** Correlation between RFU determined by SOMAscan and protein levels determined by ELISA (for ENO1 and EGF) and Immage nephelometer (for C3 and C4). **(D)** Comparison of complement levels, IFN and ESSDAI score between fatigued and non-fatigued pSS patients.

Characteristics of fatigued and non-fatigued pSS patients are summarized in Table 1. In total 16 serum proteins were differentially expressed between fatigued and non-fatigued pSS patients, of which 14 were upregulated and 2 were downregulated in fatigued patients. Top upregulated DEPs included neuroactive synaptosomal-associated protein 25 (SNAP-25), alpha-enolase (ENO1) and ubiquitin carboxyl-terminal hydrolase isozyme L1 (UCHL1). Furthermore, the proinflammatory mediator IL36a and several complement factors were upregulated in fatigued compared to non-fatigued pSS patients. A heatmap representing the DEPs is shown in Figure 2A. When unsupervised clustering of patients was performed 15 of the 22 fatigued patients (~68%) clustered together and only one non-fatigued patients clustered with this group. This grouping of fatigued pSS patients indicated a signature for fatigue in pSS. A volcano plot of the DEPs is shown in Figure 2B and a full list of all DEPs, Log Fold changes and (adjusted) *p*-values is depicted in Table 2 and Supplementary Table 5.

**Table 1:** Characteristics fatigued and non-fatigued pSS patients^*^.

	**Fatigued (*n* = 22)**	**Non-fatigued (*n* = 23)**	**Significance**
**DEMOGRAPHICS**			
Female (%)	22/22 (100)	21/23 (91)	n.s.
Mean age (years)	58.7 ± 11.3	57.3 ± 12.4	n.s.
Disease duration (years)	11.0 (15.5)	11.5 (16.8)	n.s.
ESSDAI	7.5 (8.0)	4.0 (9.5)	n.s.
IFN score	3.6 (9.4)	11.2 (8.7)	*p* = 0.020
CES-D	27.0 (18.0)	6.0 (4.0)	*p* < 0.0001
MFI			
General fatigue	20.0 (1.5)	10.0 (6.0)	*p* < 0.0001
Physical fatigue	19.0 (3.0)	8.0 (5.5)	*p* < 0.0001
Mental fatigue	17.0 (5.5)	7.0 (5.0)	*p* < 0.0001
Reduced motivation	17.0 (5.0)	6.0 (4.0)	*p* < 0.0001
Reduced activity	18.0 (4.0)	7.0 (5.0)	*p* < 0.0001
**MEDICATION STATUS (%)**			
Pilocarpine	11/22 (50)	8/23 (35)	n.s.
Hydroxychloroquine	14/22 (64)	15/23 (65)	n.s.
Corticosteroids	1/22 (5)	1/23 (4)	n.s.

*Data are presented as mean ± SD, median (IQR) or as number (%) of patients according to data distribution*.

* *Patients are selected from the cohort by a cut-off the 25 percentile highest (fatigued group) and lowest scores (non-fatigued group) in the MFI*.

*ESSDAI, the European League Against Rheumatism Sjögren's Syndrome Disease Activity Index; IFN, interferon; MFI, multiple fatigue inventory*.

**Table 2:** Differentially expressed proteins between fatigued and non-fatigued pSS patients.

	**2LogFC**	**FDR**	**Function**
**UPREGULATED PROTEIN**			
SNAP25	2.01	5.90E-04	Presynaptic plasma membrane protein involved in the regulation of neurotransmitter release. Restricted expression in brain.
C4b	1.67	1.04E-02	Basic form of complement factor 4, part of the classical activation pathway.
IL36A	1.53	1.44E-03	Cytokine that can activate NF-kappa-B and MAPK signaling pathways to generate an inflammatory response.
C3a	1.43	6.68E-03	C3a is an anaphylatoxin released during activation of the complement system.
UCHL1	1.14	2.69E-02	Belongs to the peptidase C12 family. This enzyme is a thiol protease that hydrolyzes a peptide bond at the C-terminal glycine of ubiquitin. This gene is specifically expressed in the neurons and in cells of the diffuse neuroendocrine system.
ENO1	1.00	1.10E-02	Alpha-enolase, glycolytic enzyme. Alpha-enolase has also been identified as an autoantigen in Hashimoto encephalopathy.
iC3b	0.98	4.56E-04	Proteolytically inactive product of the complement cleavage fragment C3b that still opsonizes microbes, but cannot associate with factor B.
GPD1	0.91	2.69E-02	Member of the NAD-dependent glycerol-3-phosphate dehydrogenase family. The encoded protein plays a critical role in carbohydrate and lipid metabolism.
C3d	0.69	5.60E-02	302-amino-acid fragment in the alpha chain of C3b.
BMP6	0.66	2.94E-02	Secreted ligand of the TGF-beta (transforming growth factor-beta) superfamily of proteins. Ligands of this family bind various TGF-beta receptors leading to recruitment and activation of SMAD family transcription factors that regulate gene expression.
C3	0.55	3.98E-02	Complement component C3 plays a central role in the activation of the complement system.
GOT1	0.34	2.69E-02	Glutamic-oxaloacetic transaminase is a pyridoxal phosphate-dependent enzyme which exists in cytoplasmic and mitochondrial forms, GOT1 and GOT2, respectively. GOT plays a role in amino acid metabolism and the urea and tricarboxylic acid cycles.
MAP2K1	0.30	3.98E-02	The protein encoded by this gene is a member of the dual specificity protein kinase family, which acts as a mitogen-activated protein (MAP) kinase kinase. MAP kinases, also known as extracellular signal-regulated kinases (ERKs), act as an integration point for multiple biochemical signals.
CLEC4M	0.23	4.60E-02	Involved in the innate immune system and recognizes numerous evolutionarily divergent pathogens ranging from parasites to viruses
**DOWNREGULATED PROTEIN**			
FTCD	−1.24	2.94E-02	The protein encoded by this gene is a bifunctional enzyme that channels 1-carbon units from formiminoglutamate, a metabolite of the histidine degradation pathway, to the folate pool.
EGF	−1.07	2.94E-02	Member of the epidermal growth factor superfamily.

As hydroxychloroquine (HCQ) is sometimes used to treat fatigue, differential protein expression was additionally determined after exclusion of patients who used HCQ. No differences were found compared to the analyses including HCQ users (data not shown). Additionally, dimensions of fatigue were compared between HCQ users and non-users and no differences were observed (Supplementary Figure 2).

The SOMAscan data of DEPs were validated by ELISAs when these were available. ELISAs for ENO1 and epidermal growth factor (EGF) showed significant correlations with RFUs determined by SOMAscan (Figure 2C). In addition, C3 and C4 RFUs were compared to C3 and C4 complement levels determined through routine diagnostics at the Erasmus MC, clinical chemistry lab by Immage nephelometer. Proteins selected for validation showed good correlation with protein levels determined by ELISA and Immage nephelometer. Fatigued pSS patients showed higher complement levels and lower IFN scores than non-fatigued patients (Figure 2D). There was no difference in European League Against Rheumatism Sjögren's Syndrome Disease Activity Index (ESSDAI) score, although there was a trend toward higher ESSDAI scores in the fatigued patients, which had higher scores in the articular and pulmonary domain (data not shown).

### Predictive Value of Markers for Fatigue in pSS

**Figure 3:**
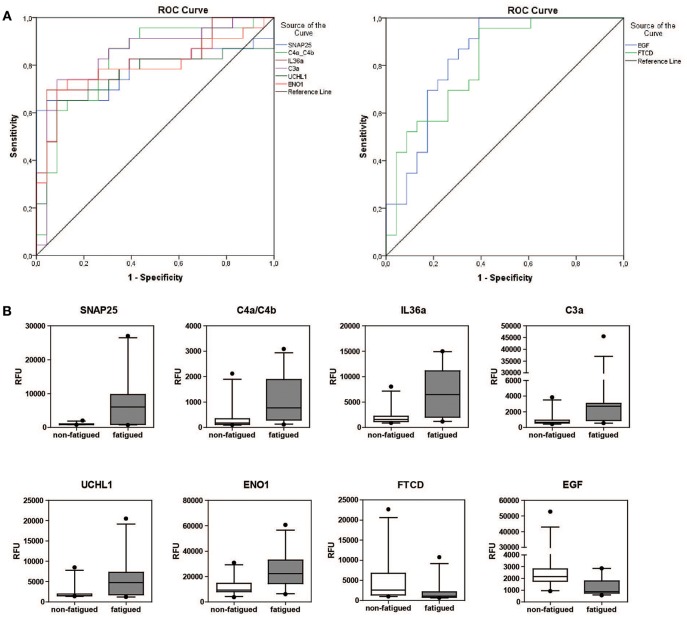
Discriminatory capacity of markers for fatigue in pSS. **(A)** ROC curves of positively and negatively predictive proteins (2LogFC>1) for fatigue in pSS. **(B)** Boxplots of differentially expressed proteins between fatigued (*n* = 22) and non-fatigued (*n* = 23) pSS patients.

The predictive potential of the proteins found to be differentially expressed between fatigued and non-fatigued pSS patients was studied. In order to do this ROC curves were calculated for the most DEPs (^2^LogFC>1) including SNAP25, complement factors C4a/C4b and C3a, IL36a, UCHL1, ENO1, EGF and formimidoyltransferase-cyclodeaminase (FTCD) (Figure 3A). Additionally, the corresponding boxplots are shown (Figure 3B). ROC analysis yielded AUC values between 0.752 and 0.845 confirming a robust discriminatory capacity between fatigued from non-fatigued patients pSS patients using these proteins (Table 3).

**Table 3:** Area under the ROC Curve for markers for fatigue in pSS.

**Test result variable(s)**	**Area**	**Std. Error^a^**	**Asymptotic Sig^b^**	**Asymptotic 95% confidence interval**	
				**Lower bound**	**Upper bound**
SNAP25	0.781	0.075	0.001	0.634	0.927
C4a_C4b	0.824	0.064	0.000	0.699	0.949
IL36a	0.819	0.065	0.000	0.692	0.945
C3a	0.845	0.061	0.000	0.724	0.965
UCHL1	0.752	0.078	0.003	0.599	0.906
ENO1	0.790	0.071	0.001	0.650	0.930
EGF	0.837	0.061	0.000	0.718	0.957
FTCD	0.811	0.064	0.000	0.686	0.936

a *Under the non-parametric assumption*.

b *Null hypothesis: true area = 0.5*.

## Discussion

PSS is a heterogeneous disease with complex pathogenesis. Traditional proteomic approaches of lachrymal or salivary fluids have shown increased expression of inflammatory and immune response-related proteins (41). Furthermore, gene expression profiling of pSS blood also revealed systemic upregulation of immune related pathways, like the IFN pathway and B cell receptor signaling pathway (18, 42, 43). Using SOMAscan technology we were able to identify upregulation of similar pathways as described using other proteomic techniques. To our knowledge one other study used SOMAscan technology to study pSS (44), although a more limited number of proteins were measured. Nishikawa et al. identified several DEPs in serum of pSS patients compared to serum of HCs and DEPs were linked to disease activity measured by ESSDAI score. When we compared pSS patients with HC we identified sets of upregulated proteins such as CD163, CXCL10, TNFSF15, FSH, CXCL11, and β2-microglobulin, that were in agreement with previously published data (44). In summary, we identified similar upregulated pathways as identified with other microarray platforms (15–19) and found similar upregulated proteins with the same technique in a different cohort of pSS patients (44) indicating the SOMAscan technology as a reliable method for the discovery of biomarkers for fatigue in pSS.

Fatigue is the most prevalent extraglandular symptom in pSS of which we do not know the biological basis. Since fatigue is often seen in conditions where the immune system is dysregulated, proinflammatory mechanisms have been thought to play a role. Previous attempts, however, to find a link between proinflammatory signatures in serum or tissue and fatigue have failed (13, 29, 45). Previous studies even showed decreasing levels of several proinflammatory cytokines like IP-10/CXCL10, TNFα, LTα, and IFNγ in fatigued pSS patients (13). Furthermore, we previously described a negative trend between IFNs and fatigue (29). In our current multiplexed proteomic analysis we show the coordinated upregulation of a set of proteins of which some are involved in inflammation including IL36a and complement factors. IL36a is a pro-inflammatory cytokine belonging to the IL-1 family and induces maturation of dendritic cells and drives Th1 and Th17 responses in CD4+ T cells (46). This cytokine was previously shown to be overexpressed in the salivary glands and serum of pSS patients (47). Upregulation of this cytokine is also seen in other diseases like psoriasis, rheumatoid arthritis, systemic lupus erythematosus, inflammatory bowel disease and fibromyalgia (46, 48, 49). In addition to IL36a, several complement factors were upregulated in fatigued pSS patients compared to non-fatigued patients. Quantification of the complement levels, however, showed that all values were in the normal range, but the non-fatigued patients lean toward reduced complement levels. Reduced complement levels are often associated with more severe disease manifestations, vasculitis, and lymphoma in pSS (50).

Interestingly, among the “fatigue-signature“ proteins were several proteins which have functions in the brain like SNAP-25, UCHL1, and ENO1. SNAP-25 protein is a SNARE protein, critical in neurotransmitter release (51). Aberrancies in this protein are described in several neurological, cognitive and psychological disorders like Alzheimer's disease and fibromyalgia (52–55). Also UCHL1 is particularly abundant in the brain, where it is critical for proper function of the ubiquitin-proteasome system in neurons (56). Reduced levels of this gene have also been linked to among others Parkinson and Alzheimer's disease (56–59). ENO1 is a glycolytic enzyme which can be expressed in the brain, but other tissues can also express this protein and it has a wide variety of functions [reviewed in (60)]. This protein has also been implicated in Alzheimer's disease. Interestingly, data indicate that ENO1 acts as an autoantigen in several autoimmune diseases. Antibodies against ENO1 have been described in Hashimoto's encephalopathy, Behçet's disease, Crohn's disease, rheumatoid arthritis (61–65) Recently, antibodies against citrullinated ENO1 (Anti-CEP-1) peptides have also been observed in pSS (66) and this raises the question if such autoantibodies associate with fatigue. Although aberrancies in all these proteins have been linked to a variety of conditions they have never been described in the context of fatigue.

EGF and FTCD were both significantly reduced in fatigued patients compared to non-fatigued patients. EGF is found in many secretions including saliva. After binding to the EGF receptor it regulates epithelial cell proliferation and survival and therefore is thought to have protective effects. EGF has previously been shown to be reduced in tears (67), salivary glands (68, 69) and saliva of pSS patients and correlates with progression of intraoral manifestations (70, 71). FTCD is a metabolic enzyme, which is primary active in the liver and kidneys. However, recently a study described additional neurological effects (72). So far none of these proteins have been linked to fatigue.

Glycerol-3-phosphate dehydrogenase [NAD(+)] (GPD1), bone morphogenetic protein 6 (BMP6), aspartate aminotransferase (GOT1), dual specificity mitogen-activated protein kinase kinase 1 (MAP2K1) and C-type lectin domain family 4 member M (CLEC4M) were additionally found slightly elevated in fatigued pSS patients compared to non-fatigued patients. These proteins have a variety of metabolic and immunological functions and GPD1, BMP6 and GOT1 also have functions in the brain. However, it is unclear how these proteins could contribute to fatigue.

Recently, proteomics performed on CSF revealed a signature for fatigue in pSS patients (73). In this abstract they describe similar as in our study upregulation of molecules in the complement system. Overall most discriminatory proteins between fatigued and non-fatigued pSS patients were involved in innate immunity, cellular stress defense and/or function in the central nervous system. It would be interesting to compare the proteins found differentially expressed in the CSF of fatigued pSS patients with the proteins we found in the serum.

A limitation of this study is that we were not able to validate all DEPs between fatigued and non-fatigued patients because there were no sensitive ELISAs available for these proteins. However, in this study we showed that when ELISAs were available, DEPs identified by SOMAscan showed good correlation with protein levels measured using different techniques indicating the reliability of the technology. Another limitation of this study is the cross-sectional design and limited number of patients in the fatigue vs. non-fatigue comparison. Furthermore, there could be underlying confounding comorbidities leading to fatigue in some patients.

## Conclusion

In this study we validated the use of aptamer-based multiplex proteomics and identified a novel set of proteins which were able to distinguish fatigued from non-fatigued pSS patients and identified a so-called “fatigue-signature.” Overall these proteins were involved in inflammatory mechanisms and have neurological and metabolic functions. More studies are necessary to validate these proteins as markers for fatigue in pSS.

## Data Availability

The raw data supporting the conclusions of this manuscript will be made available by the authors, without undue reservation, to any qualified researcher. For this the corresponding author can be contacted.

## Author Contributions

IB, MS, PK, and MV conceived and designed the study. IB, AW, and CvH-M performed the experiments. IB, LL, MS, PK, and PvdS contributed to the data analyses and interpretation. LT and PvD were involved in clinical data acquisition. IB, PvdS, and MV wrote the manuscript.

### Conflict of Interest Statement

The authors declare that the research was conducted in the absence of any commercial or financial relationships that could be construed as a potential conflict of interest.

## Supplementary Material

Supplementary Figure 1 Volcano plot of differentially expressed proteins between interferon positive (*n* = 30) and interferon negative (*n* = 30) pSS patients.

Supplementary Figure 2 Comparison of dimensions of fatigue measured by the multiple fatigue inventory questionnaire between HCQ (*n* = 40) and non-HCQ (*n* = 22) treated pSS patients.

Supplementary Table 1 Characteristics of patients and healthy controls.

Supplementary Table 2 Differentially expressed serum proteins between pSS patients and HC.

Supplementary Table 3 Characteristics of IFNpos and IFNneg pSS patients.

Supplementary Table 4 Differentially expressed serum proteins between IFNpos and IFNneg pSS patients.

Supplementary Table 5 Differentially expressed serum proteins between fatigued and non-fatigued pSS patients.
