# Supplementary figures and images for: Fatigue in Sjögren's Syndrome: A Search for Biomarkers and Treatment Targets
Source: Front Immunol. 2019 Feb 26;10:312. doi: 10.3389/fimmu.2019.00312 (PMC6399420; doi:10.3389/fimmu.2019.00312)

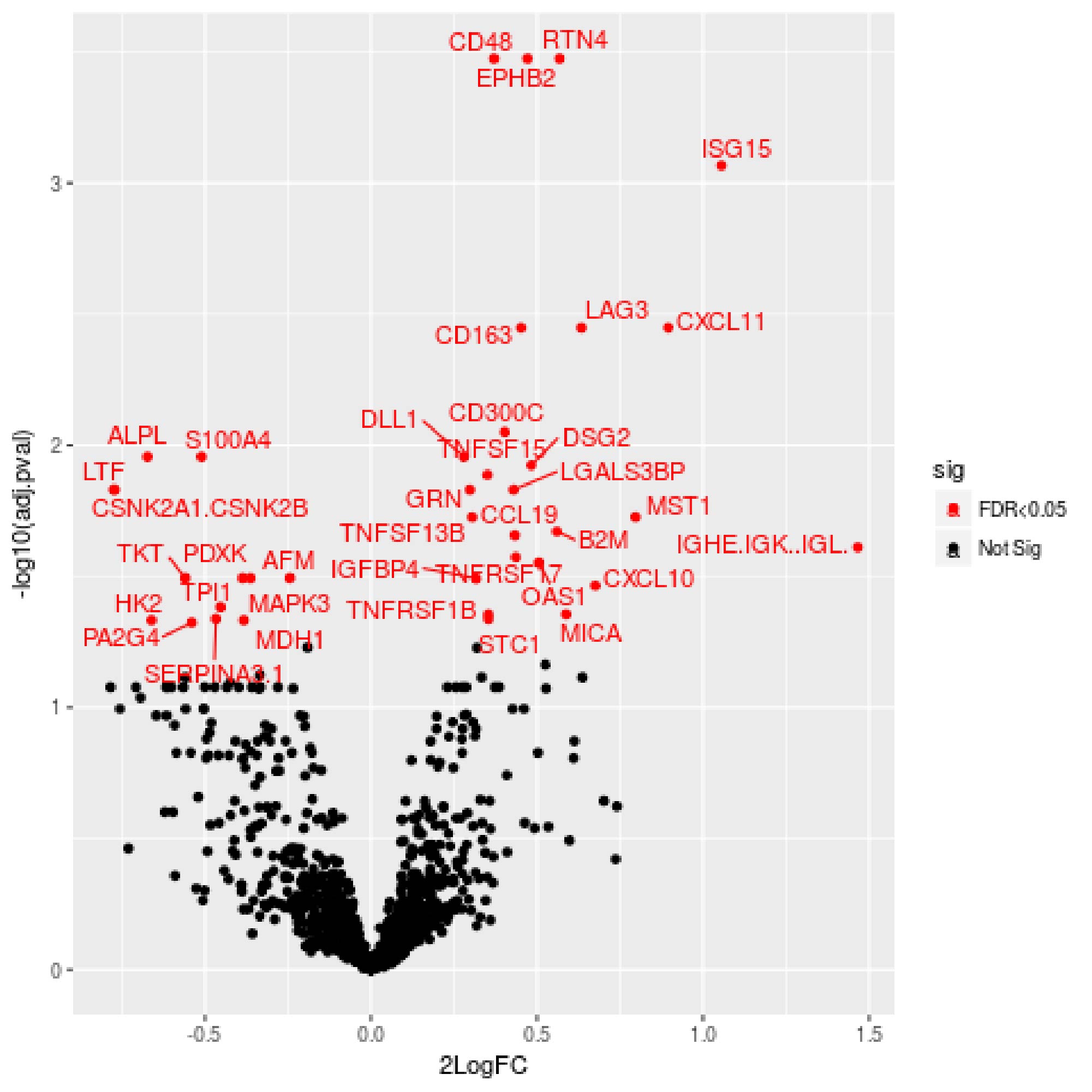

Supplement: Supplementary Figure 1 — Volcano plot of differentially expressed proteins between interferon positive (n = 30) and interferon negative (n = 30) pSS patients. [file Image_1.JPEG]

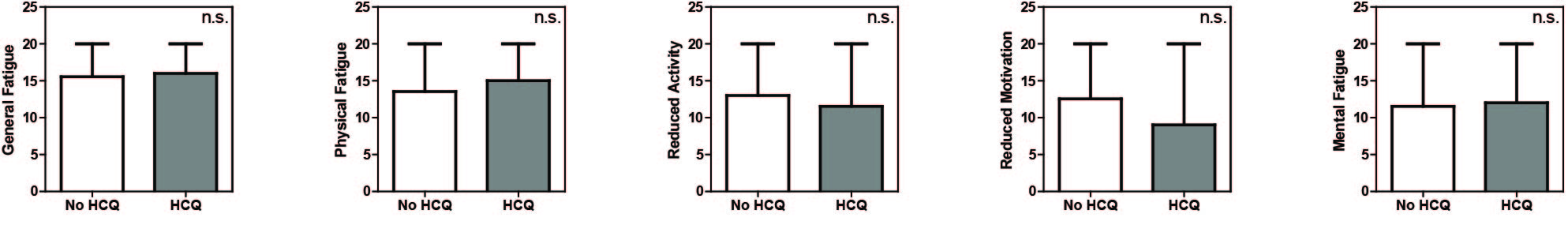

Supplement: Supplementary Figure 2 — Comparison of dimensions of fatigue measured by the multiple fatigue inventory questionnaire between HCQ (n = 40) and non-HCQ (n = 22) treated pSS patients. [file Image_2.JPEG]
