# Supplementary material for: Fatigue in Sjögren's Syndrome: A Search for Biomarkers and Treatment Targets
Source: Front Immunol. 2019 Feb 26;10:312. doi: 10.3389/fimmu.2019.00312 (PMC6399420; doi:10.3389/fimmu.2019.00312)
Supplement: Supplementary Table 1 — Characteristics of patients and healthy controls. [file Table_1.DOCX]

| **Supplementary table S1: Characteristics of patients and healthy controls** | | |
| --- | --- | --- |
|  |  |  |
|  | **HC (n=20)** | **pSS (n=63)** |
| **Demographics** |  |  |
| Female (%) | 17/20 (85%) | 59/63 (94%) |
| Mean age (years) | 35.6 ± 16.2 | 60.1 ± 12.4 |
| **Disease duration (years)** | - | 12.1 ± 8.4 |
| **Clinical manifestations** |  |  |
| Ocular symptoms | - | 63/63 (100%) |
| Oral symptoms |  | 63/63 (100%) |
| Anti-SSA positivity | - | 46/63 (73%) |
| Anti-SSB positivity | - | 31/63 (49%) |
| **ESSDAI** | - | 8.4 ± 7.0 |
| **Medication status (%)** |  |  |
| Pilocarpine | - | 26/63 (41%) |
| Hydroxychloroquine | - | 41/63 (65%) |
| Corticosteroids | - | 4/63 (6%) |
| Data are presented as mean ± SD, median (IQR) or as number (%) of patients according to data distribution. | | |
| HC, Healthy controls; pSS, primary Sjögren’s Syndrome; ESSDAI, the European League Against Rheumatism Sjögren’s Syndrome Disease Activity Index | | |
