# Supplementary material for: Fatigue in Sjögren's Syndrome: A Search for Biomarkers and Treatment Targets
Source: Front Immunol. 2019 Feb 26;10:312. doi: 10.3389/fimmu.2019.00312 (PMC6399420; doi:10.3389/fimmu.2019.00312)
Supplement: Supplementary Table 2 — Differentially expressed serum proteins between pSS patients and HC. [file Table_2.DOCX]

**Supplementary table S2: Differentially expressed serum proteins between pSS patients and HC**

| **SeqId** | **SomaId** | **TargetFullName** | **Target** | **UniProt** | **EntrezGeneID** | **Entrez**  **Gene**  **Symbol** | **2LogFC** | **FDR** |
| --- | --- | --- | --- | --- | --- | --- | --- | --- |
| **Upregulated proteins** | | | | | | | | |
| 3032-11_2 | SL000428 | Follicle stimulating hormone | FSH | P01215. P01225 | 1081 2488 | CGA FSHB | 1.56 | 0.0206 |
| 4914-10_1 | SL001766 | Human Chorionic Gonadotropin | HCG | P01215.P01233 | 1081 1082 | CGA CGB | 1.23 | 0.0241 |
| 8476-11_3 | SL002762 | Chromogranin-A | CgA | P10645 | 1113 | CHGA | 1.03 | 0.0254 |
| 14151-4_3 | SL015510 | Ubiquitin-like protein ISG15 | UCRP | P05161 | 9636 | ISG15 | 1.02 | 0.0006 |
| 3813-3_2 | SL006913 | Tyrosine-protein kinase Fyn | FYN | P06241 | 2534 | FYN | 0.88 | 0.0124 |
| 5464-52_3 | SL003792 | Growth factor receptor-bound protein 2 | GRB2 adapter protein | P62993 | 2885 | GRB2 | 0.86 | 0.0149 |
| 4250-23_3 | SL006268 | NSFL1 cofactor p47 | NSF1C | Q9UNZ2 | 55968 | NSFL1C | 0.78 | 0.0136 |
| 3310-62_1 | SL010460 | Low affinity immunoglobulin gamma Fc region receptor II-b | FCG2B | P31994 | 2213 | FCGR2B | 0.78 | 0.0206 |
| 7655-11_3 | SL002785 | N-terminal pro-BNP | N-terminal pro-BNP | P16860 | 4879 | NPPB | 0.76 | 0.0306 |
| 3420-21_2 | SL004869 | Carbonic anhydrase 13 | Carbonic anhydrase XIII | Q8N1Q1 | 377677 | CA13 | 0.73 | 0.0465 |
| 5248-68_2 | SL005793 | Peptidyl-prolyl cis-trans isomerase F. mitochondrial | Cyclophilin F | P30405 | 10105 | PPIF | 0.71 | 0.0021 |
| 3381-24_2 | SL010500 | Tyrosine-protein kinase Lyn. isoform B | LYNB | P07948 | 4067 | LYN | 0.7 | 0.0149 |
| 3038-9_2 | SL003326 | C-X-C motif chemokine 11 | I-TAC | O14625 | 6373 | CXCL11 | 0.69 | 0.0241 |
| 8484-24_3 | SL000498 | Leptin | Leptin | P41159 | 3952 | LEP | 0.69 | 0.0208 |
| 3311-27_1 | SL008609 | Low affinity immunoglobulin gamma Fc region receptor III-B | FCG3B | O75015 | 2215 | FCGR3B | 0.68 | 1.18E-06 |
| 9188-119_3 | SL003188 | C-X-C motif chemokine 9 | MIG | Q07325 | 4283 | CXCL9 | 0.63 | 0.0241 |
| 4374-45_2 | SL003869 | Growth/differentiation factor 15 | MIC-1 | Q99988 | 9518 | GDF15 | 0.63 | 0.0003 |
| 10370-21_3 | SL004396 | Signal transducer and activator of transcription 1-alpha/beta | STAT1 | P42224 | 6772 | STAT1 | 0.63 | 0.0155 |
| 3836-51_2 | SL010528 | Ubiquitin-fold modifier 1 | UFM1 | P61960 | 51569 | UFM1 | 0.62 | 0.0149 |
| 4141-79_1 | SL003183 | C-X-C motif chemokine 10 | IP-10 | P02778 | 3627 | CXCL10 | 0.62 | 0.0245 |
| 3453-87_2 | SL006917 | Tyrosine-protein kinase Lyn | LYN | P07948 | 4067 | LYN | 0.61 | 0.0316 |
| 3485-28_2 | SL000283 | Beta-2-microglobulin | b2-Microglobulin | P61769 | 567 | B2M | 0.59 | 0.0088 |
| 3397-7_4 | SL010616 | Tyrosine-protein phosphatase non-receptor type 11 | SHP-2 | Q06124 | 5781 | PTPN11 | 0.58 | 0.0175 |
| 4460-8_2 | SL006998 | 3-phosphoinositide-dependent protein kinase 1 | PDPK1 | O15530 | 5170 | PDPK1 | 0.57 | 0.0487 |
| 10365-132_3 | SL005184 | Interleukin-23 | IL-23 | P29460. Q9NPF7 | 3593 51561 | IL12B IL23A | 0.57 | 0.0149 |
| 5437-63_3 | SL001774 | Fatty acid-binding protein. heart | FABP | P05413 | 2170 | FABP3 | 0.56 | 0.0223 |
| 11089-7_3 | SL000459 | Immunoglobulin A | IgA | P01876 P01877 | 3493 3494 | IGHA1 IGHA2 | 0.56 | 0.0098 |
| 4318-12_3 | SL004940 | Tyrosine-protein phosphatase non-receptor type 6 | PTP-1C | P29350 | 5777 | PTPN6 | 0.55 | 0.013 |
| 4922-13_1 | SL003189 | C-C motif chemokine 19 | MIP-3b | Q99731 | 6363 | CCL19 | 0.54 | 0.0006 |
| 3872-2_1 | SL005679 | Translationally-controlled tumor protein | TCTP | P13693 | 7178 | TPT1 | 0.54 | 0.0206 |
| 4976-57_1 | SL013240 | Adapter molecule crk | CRK | P46108 | 1398 | CRK | 0.54 | 0.0008 |
| 3044-3_2 | SL003323 | C-C motif chemokine 18 | PARC | P55774 | 6362 | CCL18 | 0.54 | 0.0149 |
| 5028-59_1 | SL005764 | Scavenger receptor cysteine-rich type 1 protein M130 | sCD163 | Q86VB7 | 9332 | CD163 | 0.53 | 0.0008 |
| 3480-7_1 | SL008122 | Dual specificity protein phosphatase 3 | DUS3 | P51452 | 1845 | DUSP3 | 0.51 | 0.0306 |
| 9169-14_3 | SL018938 | Small ubiquitin-related modifier 3 | SUMO3 | P55854 | 6613 | SUMO3 | 0.5 | 0.0241 |
| 4496-60_2 | SL000522 | Macrophage metalloelastase | MMP-12 | P39900 | 4321 | MMP12 | 0.49 | 0.0329 |
| 4276-10_2 | SL005358 | Phosphatidylethanolamine-binding protein 1 | prostatic binding protein | P30086 | 5037 | PEBP1 | 0.46 | 0.0227 |
| 4276-10_2 | SL005358 | Phosphatidylethanolamine-binding protein 1 | prostatic binding protein | P30086 | 5037 | PEBP1 | 0.46 | 0.0213 |
| 5099-14_3 | SL005195 | Lymphocyte activation gene 3 protein | LAG-3 | P18627 | 3902 | LAG3 | 0.45 | 0.0485 |
| 5134-52_2 | SL007547 | Hepatitis A virus cellular receptor 2 | TIMD3 | Q8TDQ0 | 84868 | HAVCR2 | 0.41 | 0.0208 |
| 5128-53_3 | SL014228 | SLAM family member 6 | SLAF6 | Q96DU3 | 114836 | SLAMF6 | 0.4 | 0.0485 |
| 3073-51_2 | SL002508 | Interleukin-18-binding protein | IL-18 BPa | O95998 | 10068 | IL18BP | 0.38 | 0.0241 |
| 3040-59_1 | SL000519 | C-C motif chemokine 3 | MIP-1a | P10147 | 6348 | CCL3 | 0.38 | 0.0149 |
| 5947-90_3 | SL000598 | Thrombopoietin | Tpo | P40225 | 7066 | THPO | 0.37 | 0.006 |
| 3152-57_1 | SL001800 | Tumor necrosis factor receptor superfamily member 1B | TNF sR-II | P20333 | 7133 | TNFRSF1B | 0.35 | 0.0226 |
| 4192-10_2 | SL008039 | Alcohol dehydrogenase [NADP(+)] | AK1A1 | P14550 | 10327 | AKR1A1 | 0.34 | 0.0371 |
| 3324-51_1 | SL007674 | T-lymphocyte surface antigen Ly-9 | LY9 | Q9HBG7 | 4063 | LY9 | 0.34 | 0.0465 |
| 8480-29_3 | SL006527 | EGF-containing fibulin-like extracellular matrix protein 1 | FBLN3 | Q12805 | 2202 | EFEMP1 | 0.34 | 0.0036 |
| 2968-61_1 | SL004686 | Tumor necrosis factor ligand superfamily member 15 | TNFSF15 | O95150 | 9966 | TNFSF15 | 0.32 | 0.0206 |
| 8464-31_3 | SL012517 | R-spondin-4 | RSPO4 | Q2I0M5 | 343637 | RSPO4 | 0.32 | 0.0206 |
| 3421-54_2 | SL004635 | Tumor necrosis factor ligand superfamily member 8 | CD30 Ligand | P32971 | 944 | TNFSF8 | 0.3 | 0.0227 |
| 5301-7_3 | SL000406 | Eotaxin | Eotaxin | P51671 | 6356 | CCL11 | 0.29 | 0.0306 |
| 3045-72_2 | SL002704 | Pleiotrophin | PTN | P21246 | 5764 | PTN | 0.29 | 0.0485 |
| 3481-87_1 | SL011073 | Xaa-Pro aminopeptidase 1 | XPNPEP1 | Q9NQW7 | 7511 | XPNPEP1 | 0.29 | 0.0485 |
| 2578-67_2 | SL000038 | C-C motif chemokine 2 | MCP-1 | P13500 | 6347 | CCL2 | 0.28 | 0.0372 |
| 3503-4_2 | SL003179 | Integrin alpha-I: beta-1 complex | Integrin a1b1 | P56199. P05556 | 3672 3688 | ITGA1 ITGB1 | 0.27 | 0.0206 |
| 2783-18_2 | SL004355 | C-C motif chemokine 3-like 1 | LD78-beta | P16619 | 414062 | CCL3L1 | 0.25 | 0.0149 |
| 13114-50_3 | SL006230 | Lumican | Lumican | P51884 | 4060 | LUM | 0.23 | 0.0444 |
|  |  |  |  |  |  |  |  |  |
| **Downregulated proteins** | | | | | | | | |
| 4916-2_1 | SL000460 | Immunoglobulin D | IgD | P01880 | 3495 50802 3535 | IGHD IGK IGL | -1.98 | 0.0306 |
| 4135-84_2 | SL000461 | Immunoglobulin E | IgE | P01854 | 3497 50802 3535 | IGHE IGK IGL | -1.57 | 0.0487 |
| 4187-49_2 | SL000247 | 6-phosphogluconate dehydrogenase. decarboxylating | 6-Phosphogluconate dehydrogenase | P52209 | 5226 | PGD | -0.88 | 0.0206 |
| 3714-49_2 | SL000382 | Creatine kinase M-type:Creatine kinase B-type heterodimer | CK-MB | P12277 P06732 | 1152 1158 | CKB CKM | -0.86 | 0.0149 |
| 4149-8_2 | SL000537 | Platelet-derived growth factor subunit B | PDGF-BB | P01127 | 5155 | PDGFB | -0.72 | 0.0003 |
| 3352-80_3 | SL010288 | Carbonic anhydrase 6 | Carbonic anhydrase 6 | P23280 | 765 | CA6 | -0.7 | 0.0485 |
| 4324-33_2 | SL008516 | Cystatin-SA | CYTT | P09228 | 1470 | CST2 | -0.63 | 0.0329 |
| 3364-76_2 | SL006910 | Cathepsin L2 | Cathepsin V | O60911 | 1515 | CTSV | -0.49 | 0.0226 |
| 4125-52_2 | SL003680 | Advanced glycosylation end product-specific receptor. soluble | sRAGE | Q15109 | 177 | AGER | -0.49 | 0.0465 |
| 4499-21_1 | SL000535 | Platelet-derived growth factor subunit A | PDGF-AA | P04085 | 5154 | PDGFA | -0.48 | 0.0003 |
| 5358-3_3 | SL008574 | Osteomodulin | OMD | Q99983 | 4958 | OMD | -0.46 | 0.0417 |
| 4541-49_2 | SL014092 | Cell adhesion molecule-related/down-regulated by oncogenes | CDON | Q4KMG0 | 50937 | CDON | -0.44 | 0.0003 |
| 3303-23_2 | SL004438 | Cystatin-M | Cystatin M | Q15828 | 1474 | CST6 | -0.41 | 0.0316 |
| 3213-65_2 | SL000640 | Nidogen-1 | Nidogen | P14543 | 4811 | NID1 | -0.4 | 0.0003 |
| 5316-54_3 | SL000558 | Prothrombin | Prothrombin | P00734 | 2147 | F2 | -0.39 | 0.0189 |
| 3175-51_5 | SL006610 | A disintegrin and metalloproteinase with thrombospondin motifs 13 | ATS13 | Q76LX8 | 11093 | ADAMTS13 | -0.39 | 0.0149 |
| 2475-1_3 | SL004010 | Mast/stem cell growth factor receptor Kit | SCF sR | P10721 | 3815 | KIT | -0.38 | 0.0149 |
| 11510-31_3 | SL005699 | Apolipoprotein L1 | Apo L1 | O14791 | 8542 | APOL1 | -0.38 | 0.0005 |
| 3179-51_2 | SL009213 | Lysosomal protective protein | Cathepsin A | P10619 | 5476 | CTSA | -0.35 | 0.0378 |
| 3535-84_1 | SL004367 | Dickkopf-related protein 1 | DKK1 | O94907 | 22943 | DKK1 | -0.32 | 0.0206 |
| 3344-60_4 | SL000272 | Antithrombin-III | Antithrombin III | P01008 | 462 | SERPINC1 | -0.32 | 0.0227 |
| 3024-18_2 | SL000250 | Alpha-2-antiplasmin | a2-Antiplasmin | P08697 | 5345 | SERPINF2 | -0.32 | 0.0003 |
| 3035-80_2 | SL004354 | Interleukin-19 | IL-19 | Q9UHD0 | 29949 | IL19 | -0.32 | 0.0213 |
| 4328-2_2 | SL013490 | Brother of CDO | BOC | Q9BWV1 | 91653 | BOC | -0.32 | 0.0465 |
| 3633-70_5 | SL008193 | Nidogen-2 | NID2 | Q14112 | 22795 | NID2 | -0.31 | 0.0245 |
| 14133-93_3 | SL000145 | Interleukin-1 receptor type 2 | IL-1 sRII | P27930 | 7850 | IL1R2 | -0.31 | 0.019 |
| 5457-5_2 | SL007471 | Collectin-12 | COLEC12 | Q5KU26 | 81035 | COLEC12 | -0.31 | 0.042 |
| 2848-2_2 | SL004652 | Wnt inhibitory factor 1 | WIF-1 | Q9Y5W5 | 11197 | WIF1 | -0.31 | 0.0036 |
| 5364-7_3 | SL007336 | Protein SET | SET | Q01105 | 6418 | SET | -0.29 | 0.0076 |
| 3043-49_2 | SL000532 | SPARC | ON | P09486 | 6678 | SPARC | -0.29 | 0.0428 |
| 3365-7_2 | SL010612 | Dickkopf-related protein 4 | Dkk-4 | Q9UBT3 | 27121 | DKK4 | -0.28 | 0.0208 |
| 4562-1_2 | SL012457 | Neurexophilin-1 | NXPH1 | P58417 | 30010 | NXPH1 | -0.28 | 0.042 |
| 5846-24_3 | SL004683 | Noggin | Noggin | Q13253 | 9241 | NOG | -0.28 | 0.021 |
| 2811-27_1 | SL001995 | Angiopoietin-1 | Angiopoietin-1 | Q15389 | 284 | ANGPT1 | -0.26 | 0.0485 |
| 13112-179_3 | SL009349 | Follistatin-related protein 1 | FSTL1 | Q12841 | 11167 | FSTL1 | -0.24 | 0.0241 |
| 5491-12_3 | SL010471 | Testican-2 | Testican-2 | Q92563 | 9806 | SPOCK2 | -0.24 | 0.0347 |
| 3449-58_2 | SL004876 | Kallistatin | Kallistatin | P29622 | 5267 | SERPINA4 | -0.23 | 0.0149 |
| 5008-51_1 | SL001815 | Superoxide dismutase [Mn]. mitochondrial | Mn SOD | P04179 | 6648 | SOD2 | -0.23 | 0.0427 |
| 5328-33_37 | SL016828 | Epidermal growth factor receptor variant III | EGFRvIII | P00533 |  | EGFR | -0.22 | 0.0149 |
| 2501-51_3 | SL000055 | Cadherin-1 | Cadherin E | P12830 | 999 | CDH1 | -0.22 | 0.042 |
| 2773-50_2 | SL001717 | Interleukin-10 | IL-10 | P22301 | 3586 | IL10 | -0.22 | 0.0149 |
| 3374-49_2 | SL010499 | Tyrosine-protein kinase HCK | HCK | P08631 | 3055 | HCK | -0.21 | 0.0245 |
| 4145-58_2 | SL004359 | Neurotrophin-3 | Neurotrophin-3 | P20783 | 4908 | NTF3 | -0.21 | 0.0271 |
| 5483-1_3 | SL010467 | Repulsive guidance molecule A | RGMA | Q96B86 | 56963 | RGMA | -0.19 | 0.0485 |
| 2942-50_2 | SL000396 | Cytochrome c | Cytochrome c | P99999 | 54205 | CYCS | -0.18 | 0.042 |
| 3581-53_3 | SL000251 | Alpha-2-HS-glycoprotein | a2-HS-Glycoprotein | P02765 | 197 | AHSG | -0.16 | 0.0329 |
