# Supplementary material for: Fatigue in Sjögren's Syndrome: A Search for Biomarkers and Treatment Targets
Source: Front Immunol. 2019 Feb 26;10:312. doi: 10.3389/fimmu.2019.00312 (PMC6399420; doi:10.3389/fimmu.2019.00312)
Supplement: Supplementary Table 3 — Characteristics of IFNpos and IFNneg pSS patients. [file Table_3.DOCX]

| **Supplementary table S3: Characteristics of IFNpos and IFNneg pSS patients** | | | |
| --- | --- | --- | --- |
|  |  |  |  |
|  | **IFNpos (n=30)** | **IFNneg (n=30)** | **P-value** |
| **Demographics** |  |  |  |
| Female (%) | 28/30 (93%) | 28/30 (93%) | n.s. |
| Mean age (years) | 58.0 ± 13.7 | 61.4 ± 10.3 | n.s. |
| **Disease duration (years)** | 12.6 ± 9.4 | 11.9 ± 7.4 | n.s. |
| **Clinical manifestations** |  |  |  |
| Anti-SSA positivity | 30/30 (100%) | 14/30 (47%) | <0.0001 |
| Anti-SSB positivity | 23/30 (77%) | 7/30 (23%) | <0.0001 |
| C3 | 1.25 (0.18) | 1.12 (0.32) | p=0.033 |
| C4 | 0.23 ± 0.08 | 0.19 ± 0.07 | n.s. |
| IgG | 16.6 (6.3) | 10.4 (3.4) | <0.0001 |
| **ESSDAI** | 9.0 (8.1) | 8.2 (6.2) | n.s. |
| **Medication status (%)** |  |  |  |
| Pilocarpine | 15/30 (50%) | 10/30 (33%) | n.s. |
| Hydroxychloroquine | 18/30 (60%) | 21/30 (70%) | n.s. |
| Corticosteroids | 1/30 (3%) | 3/30 (10%) | n.s. |
| Data are presented as mean ± SD. median (IQR) or as number (%) of patients according to data distribution. | | | |
|  | | | |
| HC, Healthy controls; pSS, primary Sjögren’s Syndrome; ESSDAI, the European League Against Rheumatism Sjögren’s Syndrome Disease Activity Index | | | |
