# Supplementary material for: Fatigue in Sjögren's Syndrome: A Search for Biomarkers and Treatment Targets
Source: Front Immunol. 2019 Feb 26;10:312. doi: 10.3389/fimmu.2019.00312 (PMC6399420; doi:10.3389/fimmu.2019.00312)
Supplement: Supplementary Table 4 — Differentially expressed serum proteins between IFNpos and IFNneg pSS patients. [file Table_4.DOCX]

**Supplementary table S4: Differentially expressed serum proteins between IFNpos and IFNneg pSS patients**

| **SeqId** | **SomaId** | **TargetFullName** | **Target** | **UniProt** | **EntrezGene**  **ID** | **Entrez**  **Gene**  **Symbol** | **2LogFC** | **FDR** |
| --- | --- | --- | --- | --- | --- | --- | --- | --- |
| **Upregulated proteins** | | | | | | | | |
| 4135-84_2 | SL000461 | Immunoglobulin E | IgE | P01854 | 3497. 50802. 3535 | IGHE IGK IGL | 1.47 | 0.0245 |
| 14151-4_3 | SL015510 | Ubiquitin-like protein ISG15 | UCRP | P05161 | 9636 | ISG15 | 1.06 | 0.0009 |
| 3038-9_2 | SL003326 | C-X-C motif chemokine 11 | I-TAC | O14625 | 6373 | CXCL11 | 0.9 | 0.0036 |
| 4407-10_1 | SL005202 | Hepatocyte growth factor-like protein | MSP | P26927 | 4485 | MST1 | 0.8 | 0.0188 |
| 4141-79_1 | SL003183 | C-X-C motif chemokine 10 | IP-10 | P02778 | 3627 | CXCL10 | 0.68 | 0.0343 |
| 5099-14_3 | SL005195 | Lymphocyte activation gene 3 protein | LAG-3 | P18627 | 3902 | LAG3 | 0.63 | 0.0036 |
| 2730-58_2 | SL005199 | MHC class I polypeptide-related sequence A | MICA | Q29983 | 4276 | MICA | 0.59 | 0.0441 |
| 5105-2_3 | SL005208 | Reticulon-4 receptor | Nogo Receptor | Q9BZR6 | 65078 | RTN4R | 0.57 | 0.0003 |
| 3485-28_2 | SL000283 | Beta-2-microglobulin | b2-Microglobulin | P61769 | 567 | B2M | 0.56 | 0.0213 |
| 10361-25_3 | SL014684 | 2'-5'-oligoadenylate synthase 1 | OAS1 | P00973 | 4938 | OAS1 | 0.51 | 0.028 |
| 5071-3_3 | SL004857 | Desmoglein-2 | Desmoglein-2 | Q14126 | 1829 | DSG2 | 0.48 | 0.0119 |
| 5077-28_3 | SL007179 | Ephrin type-B receptor 2 | EPHB2 | P29323 | 2048 | EPHB2 | 0.47 | 0.0003 |
| 5028-59_1 | SL005764 | Scavenger receptor cysteine-rich type 1 protein M130 | sCD163 | Q86VB7 | 9332 | CD163 | 0.45 | 0.0036 |
| 2665-26_2 | SL004672 | Tumor necrosis factor receptor superfamily member 17 | BCMA | Q02223 | 608 | TNFRSF17 | 0.44 | 0.0267 |
| 4922-13_1 | SL003189 | C-C motif chemokine 19 | MIP-3b | Q99731 | 6363 | CCL19 | 0.43 | 0.022 |
| 5000-52_1 | SL006522 | Galectin-3-binding protein | LG3BP | Q08380 | 3959 | LGALS3BP | 0.43 | 0.0148 |
| 5066-134_3 | SL014270 | CMRF35-like molecule 6 | CLM6 | Q08708 | 10871 | CD300C | 0.4 | 0.0089 |
| 3292-75_1 | SL010450 | CD48 antigen | CD48 | P09326 | 962 | CD48 | 0.37 | 0.0003 |
| 4930-21_1 | SL005789 | Stanniocalcin-1 | Stanniocalcin-1 | P52823 | 6781 | STC1 | 0.35 | 0.0459 |
| 3152-57_1 | SL001800 | Tumor necrosis factor receptor superfamily member 1B | TNF sR-II | P20333 | 7133 | TNFRSF1B | 0.35 | 0.0441 |
| 2968-61_1 | SL004686 | Tumor necrosis factor ligand superfamily member 15 | TNFSF15 | O95150 | 9966 | TNFSF15 | 0.35 | 0.013 |
| 2950-57_2 | SL005171 | Insulin-like growth factor-binding protein 4 | IGFBP-4 | P22692 | 3487 | IGFBP4 | 0.32 | 0.0322 |
| 3059-50_2 | SL004327 | Tumor necrosis factor ligand superfamily member 13B | BAFF | Q9Y275 | 10673 | TNFSF13B | 0.31 | 0.0188 |
| 4992-49_1 | SL007173 | Granulins | GRN | P28799 | 2896 | GRN | 0.3 | 0.0148 |
| 5349-69_3 | SL006970 | Delta-like protein 1 | DLL1 | O00548 | 28514 | DLL1 | 0.28 | 0.0111 |
|  |  |  |  |  |  |  |  |  |
| **Downregulated proteins** | | | | | | | | |
| 3427-63_2 | SL007324 | Casein kinase II subunit alpha | CSK21 | P68400 | 1457 | CSNK2A1 | -0.77 | 0.0148 |
| 2780-35_2 | SL000496 | Lactotransferrin | Lactoferrin | P02788 | 4057 | LTF | -0.77 | 0.0148 |
| 2795-23_3 | SL002077 | Alkaline phosphatase. tissue-nonspecific isozyme | Alkaline phosphatase. bone | P05186 | 249 | ALPL | -0.67 | 0.0111 |
| 13130-150_3 | SL007272 | Hexokinase-2 | HXK2 | P52789 | 3099 | HK2 | -0.66 | 0.0465 |
| 4306-4_2 | SL003655 | Transketolase | Transketolase | P29401 | 7086 | TKT | -0.56 | 0.0321 |
| 4258-15_2 | SL008331 | Proliferation-associated protein 2G4 | PA2G4 | Q9UQ80 | 5036 | PA2G4 | -0.54 | 0.0475 |
| 14116-129_3 | SL004821 | Protein S100-A4 | S100A4 | P26447 | 6275 | S100A4 | -0.51 | 0.0111 |
| 2879-9_2 | SL000248 | Alpha-1-antichymotrypsin | a1-Antichymotrypsin | P01011 | 12 | SERPINA3 | -0.47 | 0.0459 |
| 4309-59_3 | SL004812 | Triosephosphate isomerase | Triosephosphate isomerase | P60174 | 7167 | TPI1 | -0.45 | 0.0414 |
| 11098-1_3 | SL007953 | Pyridoxal kinase | PDXK | O00764 | 8566 | PDXK | -0.39 | 0.0321 |
| 3853-56_1 | SL008102 | Malate dehydrogenase. cytoplasmic | MDHC | P40925 | 4190 | MDH1 | -0.38 | 0.0465 |
| 2855-49_2 | SL000409 | Mitogen-activated protein kinase 3 | ERK-1 | P27361 | 5595 | MAPK3 | -0.36 | 0.0321 |
| 4763-31_3 | SL004742 | Afamin | Afamin | P43652 | 173 | AFM | -0.24 | 0.0321 |
