# Supplementary material for: Fatigue in Sjögren's Syndrome: A Search for Biomarkers and Treatment Targets
Source: Front Immunol. 2019 Feb 26;10:312. doi: 10.3389/fimmu.2019.00312 (PMC6399420; doi:10.3389/fimmu.2019.00312)
Supplement: Supplementary Table 5 — Differentially expressed serum proteins between fatigued and non-fatigued pSS patients. [file Table_5.DOCX]

**Supplementary table S5: Differentially expressed serum proteins between fatigued and non-fatigued pSS patients**

| **SeqId** | **SomaId** | **TargetFullName** | **Target** | **UniProt** | **EntrezGeneID** | **EntrezGeneSymbol** |
| --- | --- | --- | --- | --- | --- | --- |
| **Upregulated proteins** | | | | | | |
| 13105-7_3 | SL007311 | Synaptosomal-associated protein 25 | SNP25 | P60880 | 6616 | SNAP25 |
| 2182-54_1 | SL000318 | Complement C4b | C4b | P0C0L4 P0C0L5 | 720 721 | C4A C4B |
| 14150-7_3 | SL005177 | Interleukin-36 alpha | IL-1F6 | Q9UHA7 | 27179 | IL36A |
| 2755-8_2 | SL003220 | C3a anaphylatoxin des Arginine | C3adesArg | P01024 | 718 | C3 |
| 5019-16_2 | SL002803 | Ubiquitin carboxyl-terminal hydrolase isozyme L1 | PGP9.5 | P09936 | 7345 | UCHL1 |
| 11105-171_3 | SL003650 | Alpha-enolase | Alpha enolase | P06733 | 2023 | ENO1 |
| 2683-1_2 | SL000456 | Complement C3b, inactivated | iC3b | P01024 | 718 | C3 |
| 11081-1_3 | SL007151 | Glycerol-3-phosphate dehydrogenase [NAD(+)], cytoplasmic | GPDA | P21695 | 2819 | GPD1 |
| 5803-24_3 | SL003362 | Complement C3d fragment | C3d | P01024 | 718 | C3 |
| 8459-10_3 | SL003993 | Bone morphogenetic protein 6 | BMP-6 | P22004 | 654 | BMP6 |
| 2754-50_2 | SL000312 | Complement C3 | C3 | P01024 | 718 | C3 |
| 4912-17_1 | SL000280 | Aspartate aminotransferase, cytoplasmic | GOT1 | P17174 | 2805 | GOT1 |
| 2864-2_3 | SL003793 | Dual specificity mitogen-activated protein kinase kinase 1 | MEK1 | Q02750 | 5604 | MAP2K1 |
| 3030-3_2 | SL005158 | C-type lectin domain family 4 member M | DC-SIGNR | Q9H2X3 | 10332 | CLEC4M |
| **Downregulated proteins** | | | | | | |
| 9213-24_3 | SL018900 | Formimidoyltransferase-cyclodeaminase | FTCD | O95954 | 10841 | FTCD |
| 5509-7_3 | SL000084 | Epidermal growth factor | EGF | P01133 | 1950 | EGF |
